# The six stages of the convergence of the periodic system to its final structure

**DOI:** 10.1038/s42004-023-00883-9

**Published:** 2023-05-02

**Authors:** Andrés M. Bran, Peter F. Stadler, Jürgen Jost, Guillermo Restrepo

**Affiliations:** 1 Max Planck Institute for Mathematics in the Sciences, Leipzig, Sachsen Germany; 2 Grupo de Química de Recursos Energéticos y Medio Ambiente QUIREMA, Universidad de Antioquia, Medellín, Colombia; 3 Bioinformatics Group, Department of Computer Science, Universität Leipzig, Leipzig, Sachsen Germany; 4 Interdisciplinary Center for Bioinformatics, Universität Leipzig, Leipzig, Sachsen Germany; 5 Institute for Theoretical Chemistry, University of Vienna, Vienna, State Austria; 6 The Santa Fe Institute, Santa Fe, NM USA; 7 Facultad de Ciencias, Universidad Nacional de Colombia, Sede Bogotá, Bogotá, Colombia

**Keywords:** Cheminformatics, History of chemistry

## Abstract

The periodic system encodes order and similarity among chemical elements arising from known substances at a given time that constitute the chemical space. Although the system has incorporated new elements, the connection with the remaining space is still to be analysed, which leads to the question of how the exponentially growing space has affected the periodic system. Here we show, by analysing the space between 1800 and 2021, that the system has converged towards its current stable structure through six stages, respectively characterised by the finding of elements (1800–1826), the emergence of the core structure of the system (1826–1860), its organic chemistry bias (1860–1900) and its further stabilisation (1900–1948), World War 2 new chemistry (1948–1980) and the system final stabilisation (1980–). Given the self-reinforced low diversity of the space and the limited chemical possibilities of the elements to be synthesised, we hypothesise that the periodic system will remain largely untouched.

## Abstract (web summary)

The periodic system was formulated in the 1860s by analysing order and similarities among chemical elements, but it is unclear how the exponentially growing chemical space has affected the periodic system. Here, evolution of the chemical space between 1800 and 2021 is analyzed, and it is shown that the system converged towards its current stable structure through six historical stages.

## Introduction

The periodic system (PS) was formulated in the 1860s by analysing order and similarities among chemical elements as provided by the chemical space (CS) of those times^1,2^, that is the reported chemicals up to the 1860s^3^. Order was provided by atomic weights, which were determined by finding the smallest common combining weight of a large set of compounds containing a reference element. Similarity was mainly determined on the basis of common empirical and molecular formulas, e.g. the formation of halides with equal stoichiometric coefficients^3^. At the turn of the 20th century the recognition of the atomic structure and the further developments of quantum theory led to recognise the physics underlying order and similarity^4^. The linear order is a consequence of the increasing number of electrons associated with the neutral atoms of the elements. Similarity results from splitting the electrons of each atom into core and valence ones. The latter being the drivers of the chemistry of the elements, usually encoded in their oxidation states^5^.

Despite the key role of the CS in shaping the order and similarity relationships constituting the PS, only until recently the role of this space was taken into account to assess its interplay in the emergence of the PS^3^. The expansion of the CS between 1800 and the time of the formulation of the system led the arrangement of order and similarities of the PS to converge to a backbone structure, ultimately unveiled in the 1860s. This interplay between the CS and the PS opens the question for the current status of the system, given the exponential growth of the CS^6^. Furthermore, the PS, formulated by Meyer and Mendeleev, has been adjusted but little modified to include new elements. Does that seemingly stable system still exist? To what extent has the rise of organometallic chemistry, materials science, and other areas affected its shape? Is the icon of chemistry affected by social, epistemic and technological changes such as wars, theories and the development of new chemical techniques? Those are questions we address in the current paper by computationally analysing Reaxys®, one of the largest databases of chemical information from the dawn of the nineteenth century up to date (Reaxys is a trademark of Elsevier Limited. Copyright ⓒ2023 Elsevier Limited except certain content provided by third parties). Note that although new chemicals may challenge chemical theoretical concepts, most of the known CS is explained by current quantum chemistry^5^. Therefore, it is not our aim to question the role and applicability of quantum chemistry but to analyse the effect of the vast amount of substances upon the unfolding of the relationships among chemical elements, essential for the PS.

**Fig. 1:**
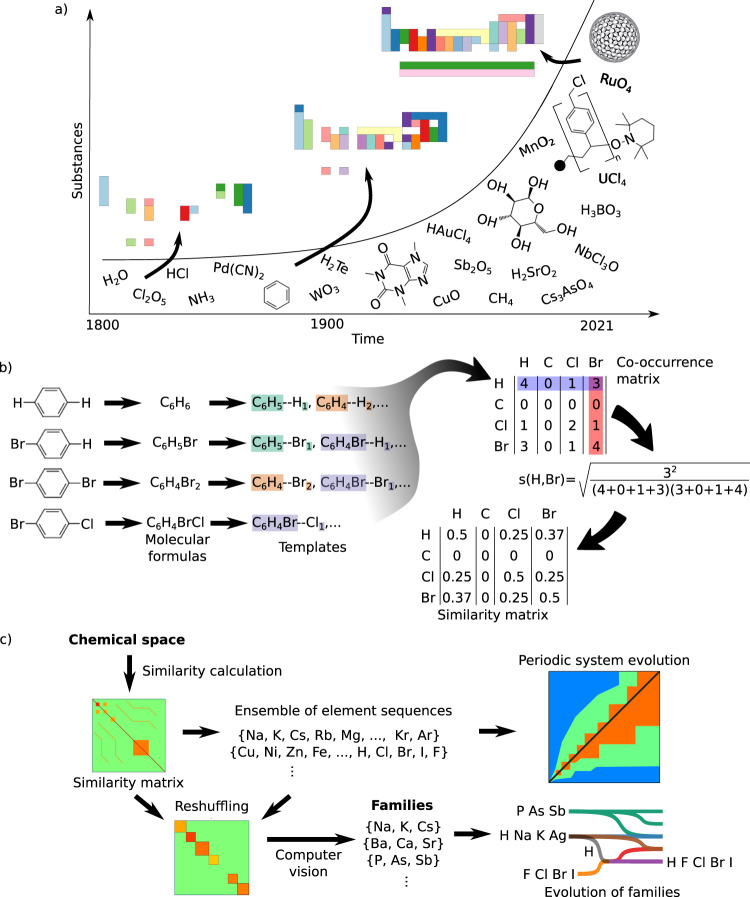
Computational approach to analyse the evolution of the periodic system (PS). **a** Chemical space (CS) expands at an exponential rate and its number and diversity of substances may affect the structure of the PS. This is here represented as possible PSs for different periods in history. **b** Calculation of element similarity as based on molecular formulas of the CS from which templates R--X_*n*_ are derived (Methods ‘Similarity between elements’). The similarity of any two elements depends on their common number of templates (co-occurrence matrix). Self-similarity is defined as the number of templates of an element contributing to similarity with other elements. **c** Calculation of similarities among PSs leading to explore the evolution of the PS as well as of its families of similar elements.

Figure 1 a schematises our aim of linking the CS with the PS. The effect of the pre-1860s space upon the system was explored in ref. ^3^. In this period, atomic debates led to different competing sets of atomic weights, which by 1860 boiled down to Cannizzaro’s weights, setting the stage for a long-sought standard set of atomic weights^7^. In^3^ was found a high correlation among all different sets of weights, including the ones currently in use. This produced largely similar order relationships between the elements, even from an early stage in history. The recognition of the relationship between atomic weight and atomic number led to take the latter as the ultimate ordering criterion for the chemical elements. Thus, the order relationship among chemical elements contained in the PS has been rather stable over the history.

In contrast, the similarity relation has not found such a stable state given its fundamental dependence on the corpus of compounds populating the CS, particularly on the stoichiometric combinations encoded in empirical and molecular formulas and on the different substance properties that may be used to asses chemical resemblance. Furthermore, the exponential expansion of the CS^6^ has been accompanied with the appearance of new chemical elements and of new combination patterns among elements, revealing in some cases new valencies; both of which have been key factors affecting the similarities among chemical elements^3^. Note that although the energetic gap separating core from valence electrons is in general wide enough to favour particular oxidation states for most of the elements, for heavy elements the gap is not that large and several electronic configurations are at the disposal of the elements, which are, in the end, determined by the bonded atoms to the elements in question^5^. Therefore, particular interests in the synthesis of some classes of compounds may favour certain oxidation states, while other interests in other periods may favour compounds with other oxidation states with repercussions upon similarity.

Aiming at studying how similarities among elements are affected by changes in the CS, here we developed a method to quantify similarity among chemical elements, further improving upon that reported in ref. ^3^, which is based on the replaceability of elements in compounds, as originally used by the formulators of the PS. We note that this is a rather general approach to chemical similarity, leaving aside particular details as those encoded in molecular structures and in the different substance properties (see Discussion below). Nevertheless, our approach based on element replaceability in formulas allows for spanning a larger CS and, therefore, to afford a general overview of the evolution of the PS.

Previous methods for quantifying similarity among elements, consider the complete replacement of one element for another in chemical formulas^3^. Thus, given the formula C_6_H_6_, to capture some similarity between H and Br, the existence of C_6_Br_6_ would be required in the CS^3,8,9^. Although valid, the complete replacement of one element for another represents only a small sample of the possible replaceabilities actually observed in substances, consequently missing important patterns leading to similarities. Our approach to similarity considers the fact that single atoms can also be independently replaced within molecular formulas, as is the case of the compounds C_6_H_6_ and C_6_H_5_Br, which differ only by substitution of one H for one Br. These are similarity patterns more common to chemist’s experience.

Figure 1b exemplifies our approach to similarity where, starting from the CS, molecular formulas of the form A_*a*_...Q_*q*_...Z_*z*_ in the dataset, are rewritten in the form A_*a*_...Q_*q*−*n*_...Z_*z*_—Q_*n*_, which makes it explicit that *n* atoms of type Q are replaceable. This leads to create templates of the form R—X_*n*_, with R representing the remaining molecular formula after extracting X_*n*_ from the original compound. The “co-ocurrence matrix” (Methods ‘Similarity between elements’) is then calculated as the number of such templates common to a pair of elements, and the similarity matrix is calculated as a normalised version of it. Introduction of new chemicals to the CS, possibly containing new elements and new (types of) substances, updates the similarity matrix. This shows how similarities are affected by the evolution of the CS and its rapid growth^6^ (Fig. 1a).

We computed similarity matrices using all chemicals available for every year in the period 1800–2021 (Supplementary Note 1). These matrices are subsequently used in various ways to represent and ultimately to gain insight into different aspects of the evolution of the PS, as shown in Fig. 1c. Aiming at contrasting the PSs of different years, we encode PSs in low dimensional representations. Research on these type of representations, and in particular in optimal sequences of elements, began in the 1980s when Pettifor introduced the first sequence and used it to visualise and estimate properties of binary inorganic compounds^10^. More recently, further approaches to the calculation of optimal sequences and extended applications have been developed^9,11^.

Using a genetic algorithm optimisation scheme similar to the one used in ref. ^9^ (Methods ‘Optimal element sequences’), we generated representative ensembles of optimal element sequences for each year between 1800 and 2021. Such sequences place similar elements in neighbouring positions within the sequence. To test the performance of our optimisation we compared them with a series of standards, including ensembles of random sequences, order by atomic number, and other previously optimised sequences^9–11^ (Supplementary Note 2, Supplementary Table 1) This also allows for assessing the performance of these sequences in our dataset, showing, e.g., that Pettifor’s scale is still one of the best performing, even after 40 years of its publication. To quantify the resemblance among PSs, we devised a similarity measure based on the relative overlapping of element sequences (Methods ‘Similarity between periodic systems (PSs)’).

In addition, to explore the evolving qualitative features of PSs (Fig. 1c), families of similar elements were automatically detected using computer vision techniques (Methods  ‘Computer vision (CV) pipeline’).

Our results demonstrate that the PS has progressed through six distinct stages, each marked by significant developments: the discovery of elements (1800–1826), the formation of the core structure of the system (1826–1860), its focus on organic chemistry (1860–1900), its further stabilisation (1900–1948), the impact of new chemistry triggered by World War 2 research (1948–1980), and the final stabilisation of the system (1980–). Due to the limited chemical possibilities for the new synthetic elements and to the self-reinforced low diversity of the chemical space, we propose that the periodic system is unlikely to undergo significant changes in the future.

## Results and discussion

### The six stages of the periodic system

**Fig. 2:**
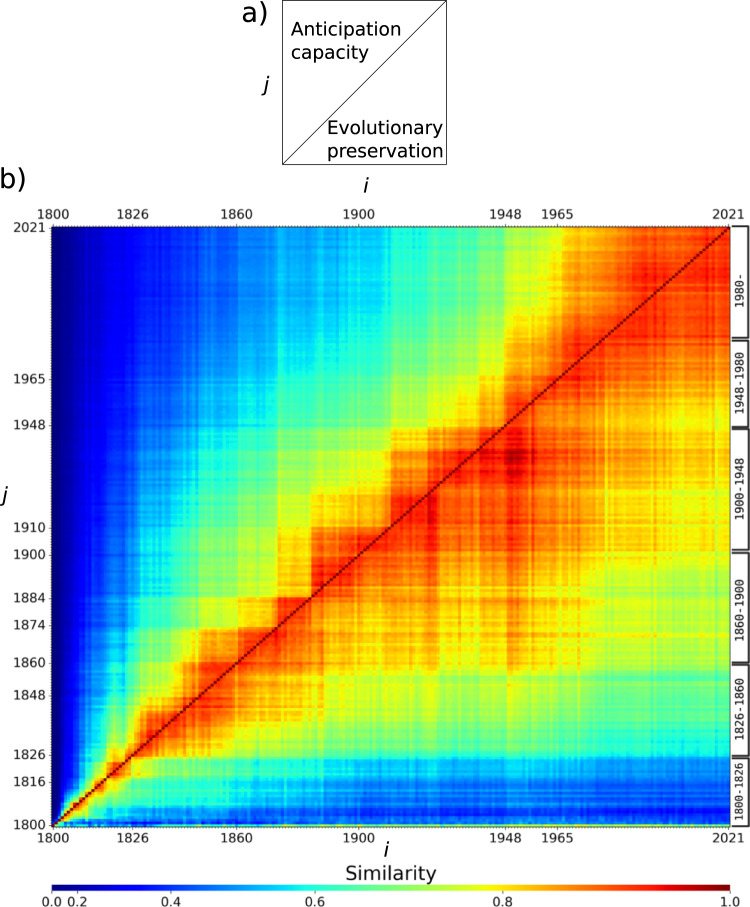
Evolution and convergence of the periodic system (PS). **a** Each value (*i*, *j*) quantifies the similarity between the PS of year *i* (column) regarding that of year *j* (row) (*z*(*i* → *j*), Methods ‘Similarity between periodic systems (PSs)’). Lower triangle indicates how much of the system of year *j* (past) remains in the system of year *i* (future) —evolutionary preservation. Upper triangle quantifies how much of the PS of year *i* (future) is in the system of year *j* (past) —anticipation capacity. **b** Divisions on the right indicate periods on the evolution of the PS, which are determined by patterns in the evolutionary preservation of the PSs they house. The unusually high values of anticipation of the PSs of 1800 are caused by the low number of elements (11) and compounds, which led to very few (13) templates (Fig. 1b) to draw similarities from, thus rendering statistics unreliable. This improved in subsequent years. Further details on high similarity values among PSs in SI Fig. 4.

**Fig. 3:**
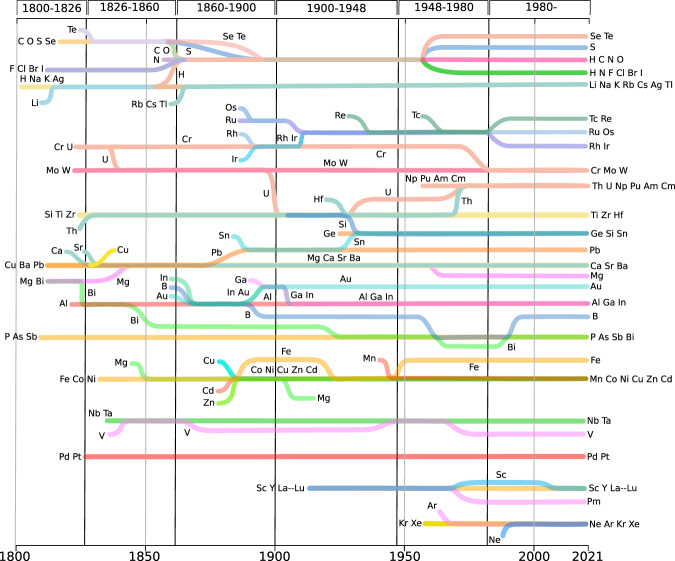
Temporal trajectories of families of similar elements. Each line represents a family that may involve one or more elements. Mergings and splittings indicate union and separation of families, respectively. Vertically, the evolutionary periods of the periodic system (PS) are shown. Mg and Cu have belonged into two families, not connected for visualisation purposes, while H and N belong simultaneously to two families after 1958. Beginning of lines have no relation to dates of discovery of elements, and are only shown whenever a given element consistently joins a family for the first time. Data supporting this figure are provided in SI Fig. 8.

**Fig. 4:**
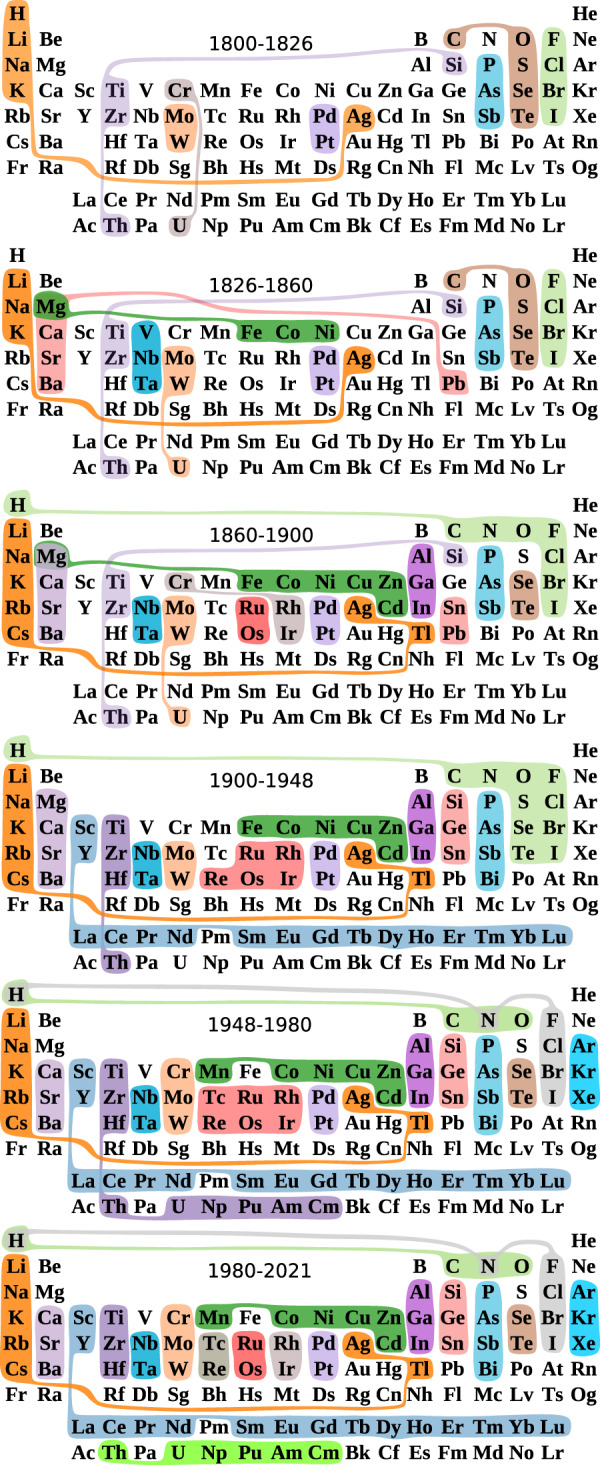
Snapshots in the evolution of the periodic system. Periodic tables representative of each period in history. Families of similar elements (sets sharing colour) shown in each table summarise the patterns shown in Fig. 3, and do not necessarily imply continuity nor simultaneity of the families throughout the period. Further details in Supplementary Note 6.

Figure 2 shows the similarities among PSs between 1800 and 2021. A key feature of this plot is the reddish region along the main diagonal, which indicates continuity in the evolution of the PS, as the most similar PS is always one of an adjacent year (SI Fig. 4). Two further aspects of the plot are relevant: its rows and columns indicate, respectively, *evolutionary preservation* and *anticipation capacity* of the PS. The former quantifies the degree of preservation of the PS of the past into the future, while the latter how much of the PS of future years is contained in systems of the past. The extension of red regions towards the right indicates high evolutionary preservation contributing to the convergence of the PS. Remarkably, this preservation does not vary monotonically, indicating the presence of some stages in the evolution of the PS, which we discuss below. In contrast, the short red regions extending upwards from the diagonal indicate that despite its convergence, the PS has undergone several updates across history making the systems of the future largely differ from those of the past. As the variation of the PS is mainly governed by similarity relations among chemical elements, rather than by their ordering^3^, the interplay of small anticipation capacities with high evolutionary preservations indicate that element similarities have been actively updated over the history of chemistry (Fig. 3) but that, nevertheless, a core of stable similarities has been often found throughout history (Fig. 4), leading ultimately to the current PS.

Notably, anticipation capacity is largely explained by changes in the number of elements (SI Fig. 9), which in turn indicates that high anticipations are possible for periods of low rates of element discovery. A more illustrative picture of the evolution of the PS is provided by evolutionary preservation patterns, which encode information about families of elements that have existed across the evolution of the CS and about their historical unfolding. That is, whether they have made their way up until the present, or whether they instead do not stand the test of time by breaking at some point (Fig. 3) Analysis of preservation patterns allows to split the evolution of the PS into six stages, shown to the right of Fig. 2.

**Fig. 5:**
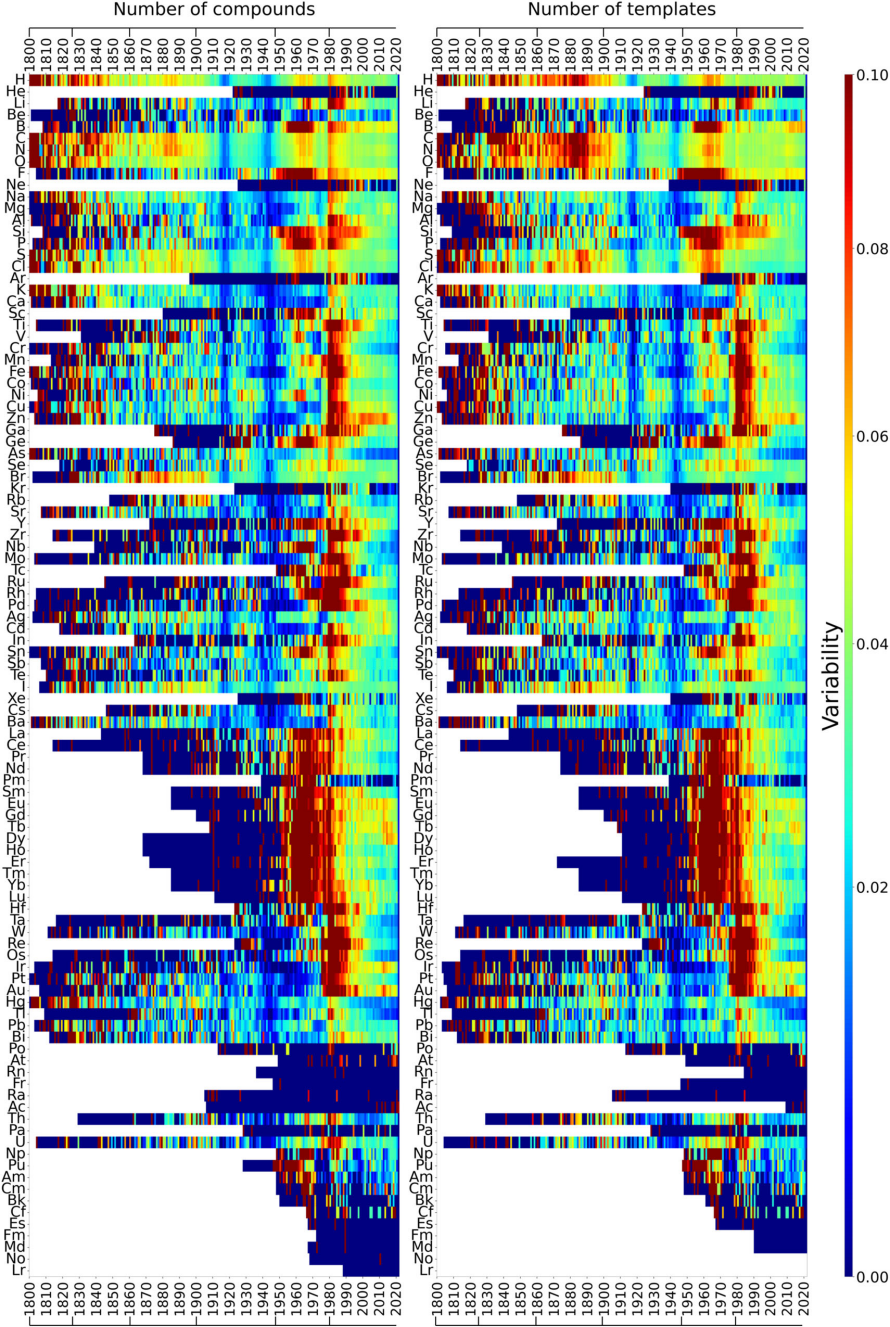
Variability in compounds and their diversity per element. Left. Variability in the number of compounds, calculated as $\log ({A}_{x,y\,+\,1})\,-\,\log ({A}_{x,y})$, where *A*_*x*,*y*_ is the accumulated number of compounds containing element *x* in year *y*. Right. Diversity of chemical formulas, calculated as $\log ({T}_{x,y\,+\,1})\,-\,\log ({T}_{x,y})$, where *T*_*x*,*y*_ is the accumulated number of templates (R–X_*n*_) (Fig. 1) found for element *x* in year *y*. Values of both plots are clipped to the range [0.0, 0.1] for visualisation purposes. As new templates correspond to previously unseen bonding patterns, the calculated difference is an indicator of compound diversity. In these plots, red indicates either a period with a surge of compounds (left) or of their diversity (right) for the element in question. Drops are indicated in blue. Dark blue bands are observed in both plots around 1915–1920 and 1940–1945 corresponding to drops caused by World Wars. Divisions at the top and bottom indicate periods on the evolution of the PS (Fig. 2).

The first stage—of setting up a basic chemical alphabet—spans the years before 1826 of highest discovery rates ever of elements (SI Fig. 9, Interactive Information). Before 1826, families of similar elements were barely preserved into the future (dark blue row at the bottom of Fig. 2) confirming the findings of^3^. The CS of those times was dominated by inorganic compounds with a growing presence of organic substances^3,6^, also evidenced in the high production of diverse metallic compounds, especially of the recently discovered alkali and alkaline-earth metals^12^ as well as in the surge of Hg, Pb, Fe and other metallic compounds and also of substances containing S, As, C and H^6^ (Fig. 5). Likewise, the chemistry of the rather reactive halogens kicked off^12^ in this period (Fig. 3), leading to the recognition of the first similarities among chemical elements and to the emergence of the oldest families of the backbone of the PS (Figs. 3, 4).

By 1826 the accelerated discovery of new elements by electrolysis and further reduction began to wind down^12,13^, which constitutes the transition to the second stage in the evolution of the PS (Fig. 2), where chemists enjoyed a rather stable set of elements that allowed for exploring their chemistries. This led to the recognition of some families of transition metals (Fig. 3). Organic chemistry surged in this period, as facilitated by advances in analytical techniques^14,15^. The growth of organic chemistry is observed in the increasing number and diversity of compounds containing organogenic elements such as H, C, N and O (Fig. 5) to the extent that H transitioned from the alkali elements to the family of organogenic elements (Fig. 3), where it has remained ever since. Alkali metals were, in turn, consolidated by including Rb, Cs and Tl, thanks to their +1-valence participation in compounds reported at that time. Valence +1 for Tl is today recognised as an evidence of the inert pair effect, explained in terms of electronic relativistic effects^16,17^. Mg, in turn, joined alkaline earths, along with Pb, and also the family Fe, Co, Ni. Nb and Ta formed a family with sporadic inclusions of V (current group 5 in the periodic table); this has remained largely untouched over the years, however V is currently a singular element. By singularity we mean the difficulty in assigning one element to one or few families, which may occur because the element has no similarity to any element, but also because of multiple ties in similarity. This shows how multiple classifications may be possible for some elements as it has been recently recognised^16^. Overall, around 65% of the PS of this period has made its way until the present (Fig. 2), and the salient structure of the PS by the time of its formulation^2,18^ is shown in Fig. 4.

About 1860 the organic chemistry side of the CS was strongly developed to the extent that it triggered the third stage of the evolution of the PS, spanning the years 1860–1900. By 1860 the semiotic capacity of the Berzelian notation—based on substance composition—saturated by the appearance of isomers^19,20^. This prompted the emergence of molecular structural theory^19,20^, which turned instrumental for a more controlled expansion of the organic chemistry side of the CS, supported by a growing synthetic activity, and by a well-established and professionalised chemistry practice with strong ties with the industry^12^. The rise of organic chemistry is evidenced in the increase of organogenic element compounds and, above all, in their diversity (Fig. 5). There was a surge of different molecular formulas made of H, C, N and O, as well as some variations of them including Cl and S^6^ (Interactive Information). This organic chemistry emphasis turned H most similar to organogenic elements, especially to Cl through one-to-one substitutions of one element for the other in organic compounds. The first decade of this period witnessed the formulation of the PS^21^, whose essential features remained almost untouched until 1970 and that even today preserves about 80% of its similarities (Figs. 2, 4). To what extent this preservation, not observed for any previous PS (Fig. 2), was important for the rise of the PS as the icon of chemistry?

The first half of the 20th century (1900–1948) corresponds to the fourth stage in the evolution of the PS, where 80% of the families are still present today (Fig. 2). In this period, chemical synthesis became the driving force for expanding the CS, which contributed to regularise the production of new chemicals by self-reinforcing the well established chemistry of the elements, where a few new valencies were discovered. This contrasts with what occurred by 1826, where new compounds made evident new combination capacities for already known elements^3^. In this period organic chemistry had a smooth growth without sudden changes in the number of compounds and their diversity (Fig. 5). Major changes in this period were on the side of some metals and other non-organogenic elements (Fig. 5). For instance compounds containing Ga, Ge and Re and some lanthanoids increased in number and diversity, which led them to form new families on the PS (Fig. 3). In particular, organic-analogous compounds started to be produced for Ge and Si after 1925, following the periodic trend encoded in the belonging of C, Si and Ge to the 14th group of the PS^22–24^. This was carried out by applying known C-reactions to Si and Ge, e.g. analogous of the Grignard reaction^22^, which led to the formation of the family {Si, Ge, Sn} (Fig. 3). In the early years of the period, S, Po and Ra increased the diversity of its compounds; while Hf, Eu, Gd and Yb increased their compounds (Fig. 5). F chemistry, in turn, gained momentum, as evidenced in the rapid increase of diverse F compounds, ranging from small representatives such as freon and UF_6_, to large compounds such as Teflon^25^. Two blue vertical lines around 1918 and 1945 in Fig. 5 indicate a drop in the diversity and number of new compounds, which demonstrate the toll of World Wars (WWs) upon chemistry. Further details on the effect of WWs on the CS are found in^6^ and its consequences for the PS are discussed below.

Unlike previous years, the PS between 1948 and 1980 (fifth stage) is largely transient, as evidenced in its low preservation values (Fig. 2). This instability is mainly caused by the appearance of new similarities among known elements such as rare earths, in particular lanthanoids, as well as among the recently discovered actinoids. Noble gases chemistry also began to be explored and the chemistry of several known elements blossomed, such as B, F and Si. Regarding rare earths, although their first compounds began to be reported at the dusk of the 18th century^26^, these elements are observed to form a family by 1912 (Fig. 3). Nevertheless, its inner structure is modified around 1952, when the chemistry of lanthanoids was further developed^25^ as motivated by WW2^26^ (SI Fig. 8). War efforts required pure uranium, which was often accompanied by rare-earth impurities^26,27^. WW2 research brought about the development of the ion-exchange separation method, which besides providing pure U samples, offered chemists, for the first time, pure samples of lanthanoids, triggering the exploration of their chemistry (see red regions around lanthanoids in Fig. 5)^27^. Actinoid chemistry also began in this period^28^ by exploring different compounds of these new synthetic elements—mainly non-carbon combinations (SI Fig. 11), which also motivated changes in some previous similarities. It is in this period, e.g., that U joined the actinoids after more than a century and a half of itinerating similarities with group-4 and -6 transition metals (Fig. 3).

Organogenic elements in this period also grew in diversity and number of compounds, and this not only occurred for the typical organic chemistry combinations of elements (CHNO, CHO, CHNOS and others reported in ref. ^6^), which spanned most of the CS of those times^6^, but also for their combination with lanthanoids (SI Fig. 12), as evidenced in the production of organometallic compounds, as well as halides and oxides^25^ (Interactive Information). Although compounds of noble gases were reported as early as 1952 for Kr and Xe^29^, the trio {Ar, Kr, Xe} became a family only by 1958 through the synthesis of some of their clathrates with formula A*2B*17H_2_O, where A is one of Ar, Kr, Xe, and B is one of the organic solvents: acetone, methyl dichloride, chloroform, and carbon tetrachloride^30^. Similarities among these elements were further strengthened by the synthesis of some of their fluorocompounds^31^ and, more recently, of their hydrofluorocompounds^32^. The inclusion of Ne occurred in 1997, through the production of atomic clusters of noble gases^33^. This is yet another example of how known periodic patterns oriented the research in the chemistry of elements.

Some individual elements prominently increased in number and diversity of compounds during this period (1948–1980), as is the case of B, F, Si, P, Sc, and Ge, showing similar patterns to organogenic elements. C compounds indeed contributed to a large extent to the revival of these element’s chemistry (SI Fig. 13), which in turn had an impact on the PS. In particular, the surge of F compounds, whose ratio of organic to inorganic compounds was above 100 by 1980, led to the splitting of the large family of H and non-metals, observed since the turn of the 20th century (Fig. 3). Thus, by 1958 this family gave place to the current fragments of chalcogens {S}, {Se, Te}; organogenic elements {H, C, N, O};, and to the parallel belonging of H to halogens and N: {H, N, F, Cl, Br, I}. The “modern age of fluorine chemistry”^34^, as it has been called the blossoming of F chemistry after WW2, involved Fowler’s perfluorination of hydrocarbons^34^, as motivated by warfare research^27,35^; Simon’s electrochemical fluorination^34^, only disclosed after WW2^27^; Fried’s report of medicinal fluorinated compounds^34^; the discovery of fluorinated noble gases^31^; and the further perfluorination methods by Margraves^34^. Thus, the role of organogenic elements, especially of C, in the chemistry of several elements in this post-war period, not only led to a surge of organogenic compounds and of their diversity (resulting from combinations with non traditional organogenic elements) but to the appearance of new similarities determining the shape of the PS in the period (Fig. 4).

The current stage began in 1980 and is characterised by the high anticipation capacities of its PSs (large red square at the top-right corner of Fig. 2), with record values of 42 years. This indicates that the PS has stayed mostly invariant since the start of this period. By 1980 there was a sharp increase in the number of compounds (Fig. 5), especially of transition metals and organogenic elements such as C, N, O, P, S, among a few others. The surge was caused in fact by organometallic chemistry (SI Fig. 10). By inspection of the molecular formulas associated with these compounds (Interactive Information), we found that they correspond to a wave of new materials, where research is focused on the physical and chemical properties of solids involving heavy metals, non-metals, and sometimes lanthanoids and actinoids^36–38^. This includes, e.g., substances developed in high-temperature-superconductor research, catalysis and some other fields^25^.

Despite the organic chemistry-guided expansion of the CS, there was also some differentiation of non-organogenic element similarities (Fig. 3), as is the case of Ru, which reached high diversity of its compounds and increased their numbers (Fig. 5). Os also enlarged the number of their compounds and Ga and In increased the diversity and number of their substances (Fig. 5). This led to the appearance of the similarity Ru-Os, as well as Ga-In, as part of {B, Al, Ga, In, Au} (Fig. 3). {Al, Ga, In} is today a family of the PS (Fig. 3). A representative periodic table of the system provided by the CS of these years is found in Fig. 4.

### On hydrogen and group-3 elements

Our results shed light on current discussions about the PS; e.g. the “correct” position of some elements such as H^39,40^. We found that those discussions must be framed in a historical context. Before mid 19th century, H was akin to alkali elements, however, once organic chemistry took the lead of the CS, H became similar to the halogens. Other discussions spin around the right constitution of group 3, with possible but excluding options: {Sc, Y, La, Ac} or {Sc, Y, Lu, Lr}. Such group-3 question even led to the creation of an IUPAC group to analyse the case and to provide a recommendation^41^. We found that there has been no moment in history depicting any of the options. CS shows a rather stable family of similar elements gathering together Sc, Y and the lanthanoids, from La to Lu, which has been well-known as rare earths for more than a century^25^. These results indicate the potential use of our data-driven methods to avoid lengthy discussions.

### The system, its perturbations and future

Our results indicate that the evolution of the PS has been mainly affected by two sorts of perturbations: changes in the number of elements and diversification of the chemistry of the elements. The former occurred, e.g. between 1800 and 1826, when the rapid discovery of elements perturbed the few similarities existing among older elements. A similar situation came about with the discovery of actinoids during WW2. Chemistry diversification, in turn, corresponds to the exploration of new facets of the chemical behaviour of formerly known elements, which we assessed in terms of the number and kind of associated chemical formulas. Diversity-triggered perturbations are exemplified by the transition of H from the alkali to the organogenic elements and by the new post-WW2 F chemistry.

Perturbations of the PS raise the question on the conditions to change the current and future PS. How likely is it to discover new elements that change the similarities supporting the PS? This is a rather unlikely event. Although further elements are expected besides the current 118 ones, new elements do not really challenge the *status quo* of the PS because their rather short lifetimes do not allow for actually exploring their chemistry as they decay before having the chance of forming compounds^42^. What about perturbations to the system by diversifying the chemistry of known elements? This requires that some elements of an existing family depict new valencies—as it was recently reported for Sc^43^—and that the number of formulas supporting these novelties surpasses those of the formulas supporting the existing similarities driven by known valencies. Achieving this requires developing new and diverse chemistry able to compete with more than two centuries of chemistry that support the current state of the PS. Although this seems unlikely given the exponential growth of the CS, doubling its size about each 16 years^6^, if the chemical community sets up to diversify the known chemistry at the historical speeds it has done it, as discussed in ref. ^44^, it may be possible in a matter of two decades to change the shape of the PS. But there are some caveats. The new chemistry should rely the least on known chemistry to avoid lengthy synthesis bringing new formulas to the CS that enlarge the set of formulas supporting the known valencies. This is the case, e.g. of compounds containing penta-valent carbons^45^. Even in such extreme, but still realistic scenario, it is very unlikely that the PS undergoes changes. A further approach to challenge the current PS involves syntheses at extreme conditions of pressure and temperature, which might unveil combination patterns not observed before for known compositions, as it has been found for NaCl with its NaCl_3_, Na_3_Cl_7_ and related compounds^46^. This approach has the advantage of not adding to the set of formulas to be challenged. Nevertheless, changing the PS through extreme conditions would require the production of new stoichiometries that compete with the more than three and a half million of different formulas reported over the history of chemistry. Extreme conditions is also an active subject of research in quantum chemistry^47^ as understanding the dramatic change on the energy gaps between core and valence electrons is of central importance for chemistry and planetary exploration.

We have shown how the PS constitutes a statistic of the CS, as it directly reflects changes in it. Therefore, the historical convergence of the system indicates a degree of preservation of the CS, that is a repetitive process in its evolution in spite of its exponential expansion. We have recently provided evidence of how the unfolding of the CS turned its evolution into a path dependent process, e.g. by the recurrent use of some few reaction classes and the frequent use of a few starting materials^48^. Rescher, in turn, has argued that repetitive science is an essential part of every science, required for attaining innovative results advancing knowledge^49^. These arguments, along with the above discussion on the unlikely challenges for the status quo of the PS, indicate that the system we currently know will likely remain as a stable object encoding the essence of a discipline gathered in the material product it creates: the CS.

### Sharpening our methods

Despite the advantages of our approach for the quantification of chemical similarity between elements, as compared to previous ones^3,8^, the method, and thus the approach to the evolution of the PS, is still based on chemical formulas. A more appropriate level of abstraction should consider molecular structure, which would allow for gauging local bonding resemblances of atoms within molecules. Although this may be considered, it also faces some challenges. In particular it lacks the generality provided by the use of molecular formulas. Similarity based on molecular structure requires information on these structures for most of the compounds and also a standard for encoding them. Popular formats include SMILES and InChIs, which assume fixed composition chemical rules, such as atom valences, whose flexibility is of primal importance for studies of this kind. Moreover, molecular structures are based on a graph-theoretical setting where bonds correspond to edges of the graph representing binary relations between atoms. Graph-theoretical models leave aside substances whose molecular representations are out of the atomic binary relationships, e.g. boranes, metallocenes and molecules holding mechanical bonds^50^. Although mathematical settings have been suggested to solve these issues^50^, these approaches need still to be further discussed. The rise of material sciences has also brought to the fore the necessity of computational standards for annotating alloys and glasses, for instance^19^. At any rate, future approaches to similarity should explicitly use the most amount of available data. We envision further similarity studies, including our approach of elements in molecular formulas, plus different further levels of chemical description of diverse complexities, ranging from elements in molecular structures, elements in chemical reactions, up to elements in reaction networks and in networks of classes of chemical reactions. Further aspects of the similarity among chemical elements, as provided by the CS, could be explored, e.g. by analysing the role of substances that are exclusive to particular elements. This approach is currently addressed by Eugenio J. Llanos, when analysing the status of this property for the current system^51^.

The convergence of the PS indicates historical patterns in the structure of the system. By using methods of time series analysis, the evolution of the PS can be further explored by retrieving details on the trends of particular similarities and their variability according to the the expansion of the CS. These approaches may therefore shed light on the memory processes along the evolution of the PS, which contributes to the understanding of the convergence of the PS as triggered by the path-dependent evolution of the CS. In this respect, it is important to analyse the rise of the PS as an organisational principle in chemistry, which may have contributed to the expansion of the space in a self-reinforced manner. That is, to what extent the PS has contributed to explore certain kind of chemistry instead of another as motivated by trying to reproduce or challenge the patterns of the PS? How far is the evolution of the PS from a self fulfilling prophecy? –to use Merton’s expression^52^. We found initial evidence of these processes, e.g. with the exploration of Si and Ge chemistries by trying to reproduce the chemistry of the members of their group on the PS. Similar approaches are followed when exploring the chemical possibilities of super heavy elements^53^.

Our approach to the evolution of the PS is entirely based on the CS. Nevertheless, other sources of information could be used to refine our results, as it was actually done by the formulators of the system. For instance, Meyer and Mendeleev, besides relying on the CS, used physical properties of the elements and their compounds. Other properties which could be important in further analyses include biological and ecological ones. At any rate, the methods used in the current work can be extended to process these properties.

Part of the success of the PS arose from its predictive power. Given the current status of the PS, the data on the CS and the computational methods provided by machine learning and artificial inteligence approaches, we are in an excellent position to undertake predictions based on the structure of the PS. This kind of approach has been reported e.g. for the prediction of enthalpies of formation of several compounds by using the conventional PS as input of a neural network model^54^.

A further question to be solved is about the role of branches of chemistry upon the PS. How does the PS look like if only inorganic substances are regarded? Is there a particular PS for material scientists, substantially different from that of organic chemistry? If this is true, what would be the implications for teaching and research?

## Methods

### Data

Substances reported in reactions, either in patent or journals, between 1800 and 2021 were dumped from the Reaxys® database^55^ on the 21st February 2022. For the resulting 18,375,580 substances, their substance ID, molecular formula (MF), and year of first publication were retrieved. As our approach to similarity relies on MFs, all isomers were collapsed into the corresponding MF; each one was assigned the ID and year of the oldest substance associated with it. This led to a dataset of 3,448,632 MFs labelled with year of publication. Further details in Supplementary Note 1. The data used in this work is property of RELX Intellectual Properties SA. The code supporting all the calculations is available at GitHub.

### Similarity between elements

Similarity between element *x* and *y* was calculated as the degree of replaceability for one another in molecular formulas (MFs). For a given set of MFs, a similarity matrix (${{\mathbb{S}}}_{t}$) collecting all pair-wise similarities among chemical elements is obtained. For the calculation, every MF of the form A_*a*_...Q_*q*_...Z_*z*_ was rewritten as A_*a*_...Q_*q*−*n*_...Z_*z*_—Q_*n*_, with *n* ≤ *q*. A set of templates R—X_*n*_ is obtained, with X being any element in the MF. In this case, it is said that element Q holds the template R—X_*n*_, as the compound R—Q_*n*_ exists in the dataset.

Similarity between elements *x* and *y* corresponds to the number *c*_*t*_(*x*, *y*) of templates they both hold in common. These values constitute the co-occurrence matrix ${{\mathbb{C}}}_{t}$. Each entry of ${{\mathbb{C}}}_{t}$ was then normalised using Equation (1)^9^. The subscript *t* emphasises the dependence of the calculation on the given CS at time *t*.

1 $${s}_{t}(x,y)\,=\,\sqrt{\frac{{c}_{t}{(x,y)}^{2}}{({\sum }_{{x}^{{\prime} }}{c}_{t}({x}^{{\prime} },y))({\sum }_{{y}^{{\prime} }}{c}_{t}(x,{y}^{{\prime} }))}}$$

Values of similarity between elements are thus bounded to the range [0, 0.5]. Equation (1) is a symmetrised version of a normalisation of the form $\frac{c(x,y)}{{\sum }_{{x}^{{\prime} }}c({x}^{{\prime} },y)}$, in which columns sum up to 1. All *s*_*t*_ values for the CS of year *t* are gathered in the similarity matrix ${{\mathbb{S}}}_{t}\,=\,{[{a}_{ij}]}_{t}$, with *a*_*ij*_ = *s*_*t*_(*i*, *j*). Instances of some of these matrices are shown in SI Fig. 1 and all matrices are available in the Interactive Information.

### Optimal element sequences

Optimal element sequences were found by optimising the cost function ${{{{{{{\mathcal{L}}}}}}}}$ (Equation (2)) over the sequence of elements *α*, given a similarity matrix ${{\mathbb{S}}}_{t}$.

2 $${{{{{{{\mathcal{L}}}}}}}}({{\mathbb{S}}}_{t},\alpha )\,=\,-\mathop{\sum}\limits_{x,y\,\ne\, x}\frac{{s}_{t}(x,y)}{{{{{{{{\rm{| }}}}}}}}\alpha (x)\,-\,\alpha (y){{{{{{{\rm{| }}}}}}}}}$$

where *α*(*x*) indicates the position of element *x* within *α*. ∣*X*∣ indicates the absolute value of *X*. ${{{{{{{\mathcal{L}}}}}}}}$ thus penalises similar elements being far from each other in the sequence. Example values of ${{{{{{{\mathcal{L}}}}}}}}$ are given in Supplementary Note 2 (Supplementary Table 1) for randomly generated sequences, as well as for previously published benchmark sequences *α*, which show incremental improvements. Likewise, SI Fig. 2 depicts some examples of cost-function values for different sequences.

Similar to^9^, the genetic algorithm used here uses the partially-mapped crossover operator^56^ as a combination scheme and mutations are introduced with a probability of 0.3. Mutations consist of moving a random slice in the sequence to another random location. After each generation, sequences are paired up based on an assigned probability proportional to a Boltzmann distribution on the cost function (Eq. (2)) with *k*_*B*_*T* = 0.7, which is scaled by 0.7 every 200 optimisation steps. Each run of the algorithm produces an initial population of 1,500 random sequences, which is evolved using the crossover and mutation operators described above. A total of 600 generations is used for each optimisation and the best overall individual (lowest value of ${{{{{{{\mathcal{L}}}}}}}}$) is selected as the result. Further details are provided in Supplementary Note 2 (Supplementary Table 1).

### Similarity between element sequences

For an optimal element sequence *α* (Methods ‘Optimal element sequences’), the set *B*_*α*_ = {{*x*, *y*}: ∣*α*(*x*) − *α*(*y*)∣ ≤ *r*} is obtained, which contains all pairs of elements *x*, *y* located at distances no more than *r* in *α*. The similarity of sequence *α* regarding ${\alpha }^{{\prime} }$ is computed as $z(\alpha \,\to\, {\alpha }^{{\prime} })\,=\,\frac{{{{{{{{\rm{| }}}}}}}}{B}_{\alpha }\cap {B}_{{\alpha }^{{\prime} }}{{{{{{{\rm{| }}}}}}}}}{{{{{{{{\rm{| }}}}}}}}{B}_{\alpha }{{{{{{{\rm{| }}}}}}}}}$. Likewise, $z({\alpha }^{{\prime} }\,\to\, \alpha )\,=\,\frac{{{{{{{{\rm{| }}}}}}}}{B}_{\alpha }\cap {B}_{{\alpha }^{{\prime} }}{{{{{{{\rm{| }}}}}}}}}{{{{{{{{\rm{| }}}}}}}}{B}_{{\alpha }^{{\prime} }}{{{{{{{\rm{| }}}}}}}}}$. ∣*X*∣ indicates the number of elements in *X*. Examples of the calculation are given in Supplementary Note 3 and further information regarding the choice of *r* is found in SI Fig. 3.

### Similarity between periodic systems (PSs)

Each PS is represented by an ensemble of optimal element sequences (Methods ‘Optimal element sequences’), which correspond to the 15 best sequences obtained from a pool of 50 parallel optimisations. To compare PSs *i* and *j* in such a format, the average similarity between all (15 × 15 = 225) pairs of sequences in the corresponding ensembles is computed:

3 $$\bar{z}(i\,\to\, j)\,=\,\frac{\mathop{\sum }\nolimits_{l,m \,\,= 1}^{15}z({\alpha }_{l}^{i}\,\to\, {\alpha }_{m}^{j})}{225\,\cdot\, z{(\cdot \to j)}_{\max }}$$

where $z({\alpha }_{l}^{i}\,\to\, {\alpha }_{m}^{j})$ corresponds to the similarity between ${\alpha }_{l}^{i}$, the *l*-th sequence of the system of year *i*, regarding the *m*-th sequence of the system of year *j*, ${\alpha }_{m}^{j}$. $z{(\cdot \to j)}_{\max }$ is a normalisation factor (highest value of column *j*). Thus, $0\,\le\, \bar{z}(i\,\to\, j)\,\le\, 1$.

### Detection of families of elements

Families of similar elements are detected using a pipeline comprising computer vision algorithms for square detection in images (CV), and a custom statistical noise reduction algorithm (SNR). This pipeline exploits the structure of re-shuffled similarity matrices and leads to reproducible and parameter-independent results. The pipeline takes a similarity matrix and an ensemble of optimised sequences as input, and outputs a collection of families of elements. In the first step, 15 images are produced from reshuffling the similarity matrices, each using one of the sequences of the ensemble. For each image, CV is applied with *N* different sets of parameters, each yielding a collection of families. SNR is then applied over the results of each image, reducing the results from the previous step to a single collection of families per image. SNR is applied once again over these resulting collections, producing a single collection of families which is the output of the pipeline. Further information is found in SI Fig. 5 and in Supplementary Notes 4 and 5.

### Computer vision (CV) pipeline

A computer vision pipeline was assembled, whose purpose is to detect square-shaped regions of relative high contrast lying on the diagonal of reshuffled similarity matrices (SI Fig. 2).

SI Fig. 6 shows a step-by-step visualisation of an example computation. CV consists of (a) replacement of the values on the diagonal by the average value of the pixels immediately to the left and right to shadow the overly high values in this zone which obscure the observation of square-like shapes. In (b), standard procedures of up-sampling, blurring and padding are applied to remove noise and increase resolution of the images as a preparation for next step. In (c), Canny’s algorithm for detecting edges is applied^57^, and in (d) shapes are detected on the resulting images. These shapes are then filtered for squares lying on the diagonal, whose size corresponds to no more than 20 elements, that is 20 pixels in the original unprocessed matrix. The method depends on four parameters in total and the following assignments were found to provide good results: up-sampling factor = 15, Blurring window size any of {17, 19, 21, 23}, and two parameters associated with Canny’s algorithm, which set the different thresholds as explained in^57^ are chosen so that *th* + *b* = 40, and *th* is sampled with a probability given by a Boltzmann distribution on *th* with parameter *β* = 20.

### Statistical noise reduction (SNR) algorithm

SNR was deviced to extract the most statistically relevant results out of a pool of collections of element families. It was used to leverage the results from applications of CV (Methods ‘Computer vision (CV) pipeline’) with various sets of parameters, as well as those stemming from reshuffling similarity matrices with the different sequences in the corresponding ensemble. As shown in SI Fig. 7, SNR takes as input a pool of collections of families, and outputs a single collection. In step a each family is expanded into a new collection, by gathering the most similar family in every other input collection (using Tanimoto similarity^58^). In b each of these is again reduced to a single family, containing the elements present in at least 50% of the families in this collection; this results in a new pool of collections, and this process is repeated M times using this result as the input (step d). In step e the output pool of collections is collapsed into a single collection. After each step, duplicates, empty, or one-element families are removed (steps c and e). The resulting collection is given as the output.

This algorithm was designed to preserve the diversity of the families produced by CV, which is achieved in a by giving each family in the input pool of collections the opportunity to prove their statistical significance by finding sufficiently similar families in other collections. Additionally, it suppresses noise by generating a new family with the most popular elements in the collection of most similar families.

## Supplementary information

Supplementary Information

## Data Availability

The data used in this work is property of RELX Intellectual Properties SA.

## Code availability

The code supporting all the calculations is available as a GitHub repository: github.com/doncamilom/Reaxys_PS.
