# Supplementary material for: The six stages of the convergence of the periodic system to its final structure
Source: Commun Chem. 2023 May 2;6:87. doi: 10.1038/s42004-023-00883-9 (PMC10154405; doi:10.1038/s42004-023-00883-9)
Supplement: Supplementary file 1 — Supplementary Information [file 42004_2023_883_MOESM1_ESM.pdf]

The six stages of the convergence of the periodic system to its final structure

Andrés M. Bran[1,2,3], Peter F. Stadler[1,3,4,5,6,7], Jürgen Jost[1,6] and Guillermo Restrepo[1,4†]

[1] Max Planck Institute for Mathematics in the Sciences, Inselstraße 22, Leipzig, 04103, Sachsen, Germany.
[2]Grupo de Química de Recursos Energéticos y Medio Ambiente QUIREMA, Universidad de Antioquia, Medellín, Colombia.
[3]Bioinformatics Group, Department of Computer Science, Universität Leipzig, Härtelstraße 16-18, Leipzig, 04107, Sachsen, Germany.
[4]Interdisciplinary Center for Bioinformatics, Universität Leipzig, Härtelstraße 16-18, Leipzig, 04107, Sachsen, Germany.
[5]Institute for Theoretical Chemistry, University of Vienna, Währingerstraße 17, Vienna, 1090, State, Austria.
[6]The Santa Fe Institute, Hyde Park Rd., Santa Fe, 1399, New Mexico.
[7]Facultad de Ciencias, Universidad Nacional de Colombia, Sede Bogotá, Av. Carrera 30 # 45-03, Bogotá, Colombia.

†Corresponding author: restrepo@mis.mpg.de

Supplementary Information

Supplementary Note 1: Data

Reaxys® database [1] was used, which comprises the different editions of the famous *Gmelins Handbuch der anorganischen Chemie* and *Beilsteins Handbuch der organischen Chemie*, along with the Patent Chemistry Database, all in digital form [2], constituting one of the largest and most complete databases of chemical knowledge to date, featuring historical labels. An extensive study on the growth of the CS is available at [3], which exposes a variety of important

features, such as the exponential growth in the number of substances with time and trends in most used compositions, among others. A curated dataset was produced from Reaxys®, which contains the following three fields: substance ID, molecular formula (MF), and year of first publication.

Reaxys distinguishes between Reaction data and Substances data, which are independent in the database but linked through identification numbers. Years of publication (and thus historical labels) are reported mainly in the Reaction side, while MFs are reported on the Substances side only. As such, both sides of Reaxys were dumped, the relevant fields were obtained from each, and then the results merged into a single file.

We considered all substances reported in reactions, either in patent or journals, between the years 1800 and 2021. Except for the removal of non-stoichiometric compounds, and substances containing the element "Pol" (referring to polymeric units), no additional filter was placed on the types of substances to be used. The final dataset contained information for 18,375,580 substances.

As the present work relies only on MFs, the dataset was further compressed by collapsing all substances sharing a common MF —isomers— into a single MF; the publication date and ID number of the oldest isomer associated with it was used. Such a setting leaves a total of 3'448.632 different MFs, which is the final dataset used in this study.

Supplementary Note 2: Optimisation with Genetic Algorithms (GA)

To test the performance of the algorithm described in Methods (Optimal element sequences), four benchmark sequences were scored and compared with our results. A comparison of the performance of the different benchmarks is given in Supplementary Table 1, which includes a) a sample of randomly generated sequences, b) ordering by atomic number (Z), c) Pettifor's scale [7], d) Glawe et al.'s GA optimised scale [8], e) Glawe et al.'s modified Pettifor scale [8] and f) Allahyari and Oganov's Universal Sequence of Elements (USE) [9]. Cost $\mathcal{L}$ was calculated using the similarity matrices $\mathbb{S}$ for 1980, 2016 and 2021, and results of our optimisations for each year are provided in Supplementary Table 1, row g. These years were selected based on the publication years of the sequences by Pettifor [7], Glawe et al. [8] and Allahyari and Oganov [9].

Pettifor' sequence [7], even with the limited dataset and methods used by the time of its formulation in 1980, improves substantially with respect to Z, and performs well afterwards, achieving comparable results to Glawe et al.' sequences, which further improve Pettifor's but not substantially. Oganov et al.' sequence [9] shows slightly better performance than Z, although far below Pettifor or Glawe et al.'s. Our optimised sequences substantially improve $\mathcal{L}$ relative to these baselines for each of the years considered, which validates their use as suitable representations of the PS.

A key difference with Glawe et al.' sequences [8], is that the endpoints of their sequences (regarded there as orderings) are fixed to be H and Kr

SI Fig. 1 Similarity matrices between elements over the years. Selected examples of similarity matrices, with rows and columns sorted according to atomic number. The obtained similarity matrices are highly structured objects containing blocks around the main diagonal, as well as diagonal and vertical/horizontal patterns. The former correspond to similarities among neighbouring elements belonging in the same period of the periodic table. For instance C, N, O and F; or Mn, Fe, Co, Ni, Cu and Zn; and the lanthanoids (from La to Lu). Diagonal patterns show similarities among elements belonging in the same family, traditionally depicted as elements of a column (group) on the conventional periodic table. Exceptions to the over-simplistic group-period characterisation of the periodic system (PS) [2, 4] are shown, e.g. the similarity between Mg and Zn, which belong to current group 2 and 12, respectively. The closest diagonal patterns to the main diagonal correspond to neighbouring elements belonging to adjacent periods on the PS, from 3rd to 6th; that is, there are similarities among 3rd and 4th period elements, 4th and 5th, etc. Similarities among 2nd and 3rd period elements are much lower, which evidences the validity of the singularity principle of second period elements [5]. A second diagonal pattern is observed farther from the main diagonal and corresponds to similarities between 3rd and 5th periods (secondary periodicity [6]). This is the case of the similarities between Mg-Cd and Al-In, for instance. Horizontal/vertical patterns are caused by the resemblance of lanthanoids regarding other elements. This is the case of their similarities with Y and Sc, which constitute the rare earths together with the lanthanoids (La-Lu). They also show some similarities with Al, Ga and In (yellow horizontal lines on top of the block), which share the typical +3 valency of lanthanoids.

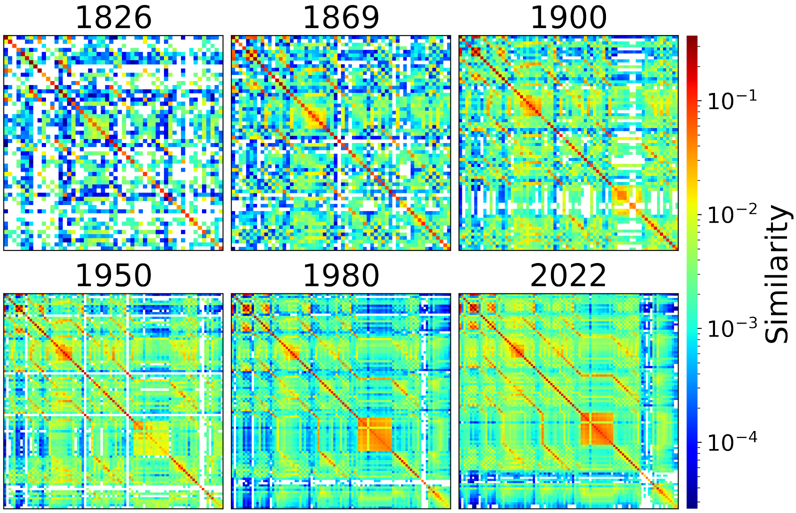

during optimisation, a decision which allowed the authors for an easier visual inspection and comparison against Pettifor's [7]. As the focus here is obtaining

SI Fig. 2 Reshuffling of similarity matrix of year 2021 as ordered by atomic number Z and three optimised sequences. Elements arranged by (**left**) atomic number and (**rest of the row**) by optimised sequences of elements (Methods - Optimal element sequences). As can be seen, the search for optimal element sequences groups together highly similar elements into blocks with high contrast relative to the background. This result motivates the use of computer vision algorithms (Methods - Computer vision) for detecting families of elements. It is also illustrated how different optimisations, for the same similarity matrix, often produce in different images, nevertheless with similar local structures, that is, roughly the same blocks are found, although in different positions within the matrix. The complete set of matrices and results of the optimisations is provided in the http://mchem.bioinf.uni-leipzig.de:8080/.

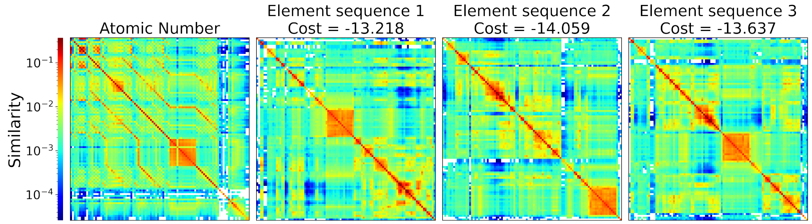

	Sequence (α)	$\mathcal{L}$ ($\mathbb{S}_{1980}$)	$\mathcal{L}$ ($\mathbb{S}_{2016}$)	$\mathcal{L}$ ($\mathbb{S}_{2021}$)
a	Random	-4.61 ± 0.24	-4.36 ± 0.23	-4.31 ± 0.23
b	Atomic number (Z)	-8.86	-8.65	-8.63
c [7]	Pettifor	-13.20	-12.77	-12.69
d [8]	Glawe et al.	-13.31	-12.86	-12.78
e [8]	Glawe et al. modified	-13.11	-12.7	-12.61
f [9]	Oganov et al.	-9.40	-9.02	-8.98
g	This work	-14.42 to -14.73	-13.66 to -14.12	-13.71 to -14.24

Supplementary Table 1: Cost function $\mathcal{L}$ of different optimal element sequences (α) under chemical spaces (CSs) ($\mathbb{S}_i$) contemporary to the publication year of the sequences. The lower the values, the better the sequences representing the periodic system of the corresponding CS.

unbiased, lower dimensional representations of a more general system, our approach makes, in contrast, a fully unconstrained optimisation of the sequence of elements.

Supplementary Note 3: Quantitative comparison of sequences of elements

Let $\alpha = \{$Ca, Ba, Na, K, Li, H, Cl, Br, I, F$\}$, and $\alpha' =\{$Li, H, Na, Ba, K, Ca$\}$; note that they have different amount of elements (10 and 6, respectively), which illustrates how sequences with different sizes can be compared.

For α, the following pairs of elements satisfy the condition of being at a distance $r \leq 2$, which are gathered in B_α=$\{\{$Ca, Ba$\}$, $\{$Ca, Na$\}$, $\{$Ba,

Na}, {Ba, K}, {Na, K}, {Na, Li}, . . .}. Likewise, $B_{\alpha'}$ is obtained, leading to $z(\alpha \to \alpha') = \frac{|B_\alpha \cap B_{\alpha'}|}{|B_\alpha|} = 6/17 = 0.35$ and $z(\alpha' \to \alpha) = 6/9 = 0.67$ (Methods - Similarity between element sequences). This indicates that only 35% of the neighbour pairs in α are also found in α'. Conversely, 67% of the pairs in α' are also found in α. This explains the huge differences between the upper and lower triangles of Fig. 2 (Main text).

SI Fig. 3 Similarity between element sequences correlates well with difference in cost $\mathcal{L}$. Correlation between difference in $\mathcal{L}$, and similarity between element sequences (Methods - Similarity between element sequences). Each column corresponds to a different value of r. A bag of sequences was built from all element sequences obtained from the optimisations (Methods - Optimal element sequences), for a total of 11,000. **Top row** compares the best obtained sequence overall, against all others. **Bottom row** compares a randomly chosen sequence, against all others. Each column corresponds to a different value of r. The parameter r used in the computation of similarity between sequences of elements (Methods - Similarity between element sequences) corresponds to a radius that defines a neighbourhood around each element in the sequence. The higher the values of r, the more loose the comparison becomes as the neighbourhoods become larger. In the limit when r approaches half of the number of elements in a sequence, all possible sequences are equal under this quantity. Thus low values of r are desired, to evaluate how local structures (in small neighbourhoods of each element) are preserved from one sequence to the other. The plot shows that r=2 is already a good choice, for which $r = 2$ is used for the computations in this work.

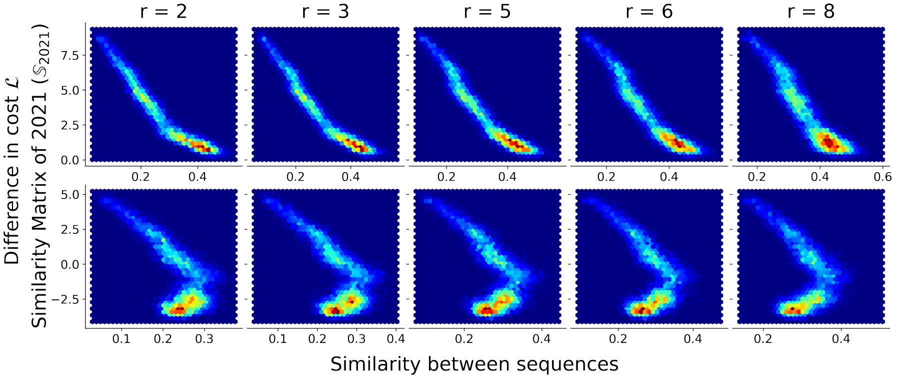

Supplementary Note 4: Detection of families of elements

Families of elements are reported as the result of combining a computer vision-based pipeline and a statistical noise reduction algorithms, which take as input a similarity matrix and a corresponding ensemble of optimised element sequences to produce a collection of families of similar elements (Methods -

SI Fig. 4 Historical convergence of the periodic system (PS). Each value quantifies the similarity between the PS of year i (column) regarding that of year j (row) ($z(i \to j)$, Methods - Similarity between PSs). Any value above a threshold of 0.88 is shown in red to highlight the strong similarity between PSs of consecutive years, showing the continuity in the evolution of the PS.

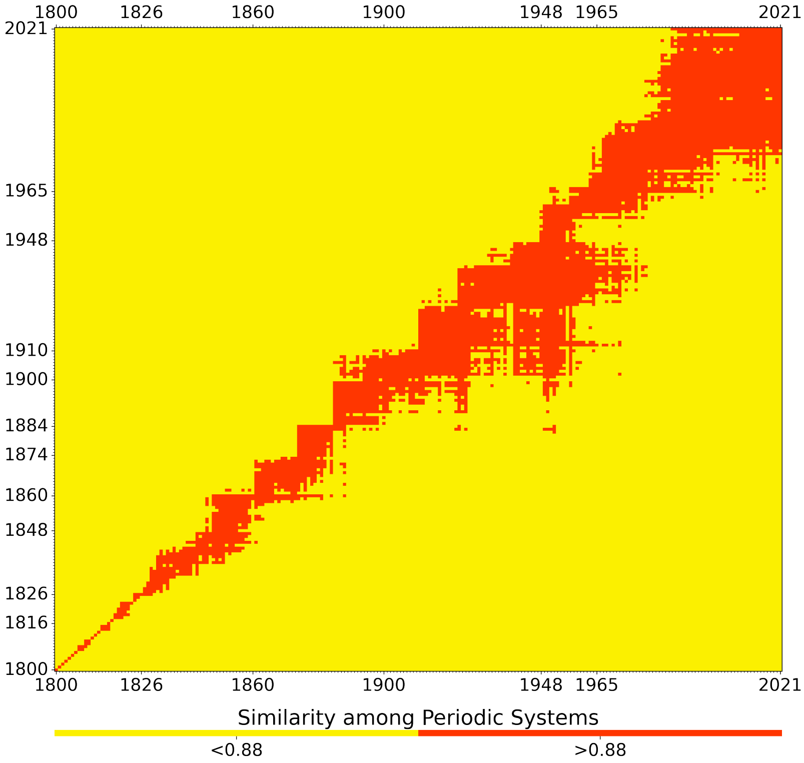

Detection of families of elements, - Computer vision and - Statistical noise reduction).

Supplementary Note 5: Computation of statistical noise reduction

The algorithm has been devised in such a way that its application allows for decreasing variability in the content of sets of elements, while preserving diversity of families. To illustrate this, consider the following three collections of families, which are given as input:

- {Mg, Ca, Sr} , {Li, Na, K, Cl}
- {Mg, Ca, Sr} , {Li, Na, K}
- {Mg, Ca, Sr, Hg} , {Li, Na, K}

SI Fig. 5 Calculation of families of elements from a similarity matrix. The method exploits the rich structure of similarity matrices when reshuffled according to the optimised sequences of elements of Methods - Optimal element sequences, through the use of a computer vision pipeline algorithm for detecting squares in images (Methods - Computer vision). These results are then processed using a Statistical Noise Reduction algorithm (SNR, Methods), that outputs the most representative families of elements, given the results from multiple runs of the computer vision pipeline. In the first step, the similarity matrix is reshuffled using each of the corresponding optimised sequences, which produces one image per sequence. For each image, CV is applied with N different sets of parameters; each application produces a collection of families of elements, thus producing a pool of N collections per image. In the next step, SNR is applied to each of these pools, producing a single collection, thus at this point there are 15 collections, one per image. Finally, this last pool of collections is again given as input to SNR, which yields a single collection of families of elements as the output.

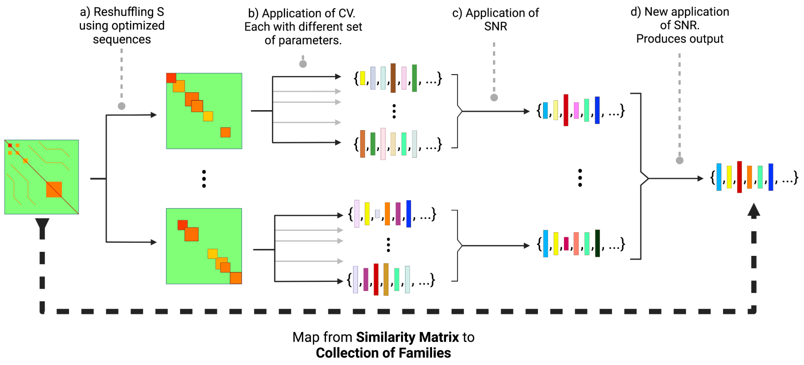

The ideal output would be the collection {{Mg, Ca, Sr} ,{Li, Na, K}}, as the elements in both of these families are predominantly found together in the families of the input collections. Variations on these (for instance inclusion of Cl or Hg) are regarded as random noise, introduced in any of the previous steps, that is in the optimisation of sequences of elements (Methods - Optimal element sequences), or in the computer vision pipeline (Methods - Computer vision). By decrease of variability we mean the removal of such noise. It is however also desired to preserve both families, in what is regarded here as preserving diversity.

The algorithm allows just that. Diversity is preserved in the first step by explicitly giving every candidate family the chance to prove their statistical significance, by gathering the most similar other families from other collections. The second step then homogenises such families, thus decreasing variability. The whole process is then repeated to ensure optimal homogenisation.

As a meta-result, we found that a single run of the algorithm (on reshuffling with a single sequence, for a given similarity matrix, step c in SI Fig. 5) results

SI Fig. 6 Example of application of the computer vision (CV) algorithm for square detection. The original similarity matrix (year 2021) is reshuffled according to an optimised sequence of elements. Diagonal smoothing is used, in which the diagonal of the matrix is hidden to better show the square patterns. This is followed by standard up-sampling, blurring and padding of the image, to increase the resolution and remove the sharp edges coming from individual pixels in the original similarity matrix. Finally, edges are detected on the resulting image using Canny's algorithm, and square shapes are detected. These shapes are filtered, so that final results are squares, lying on the diagonal, whose size corresponds to no more than 20 elements.

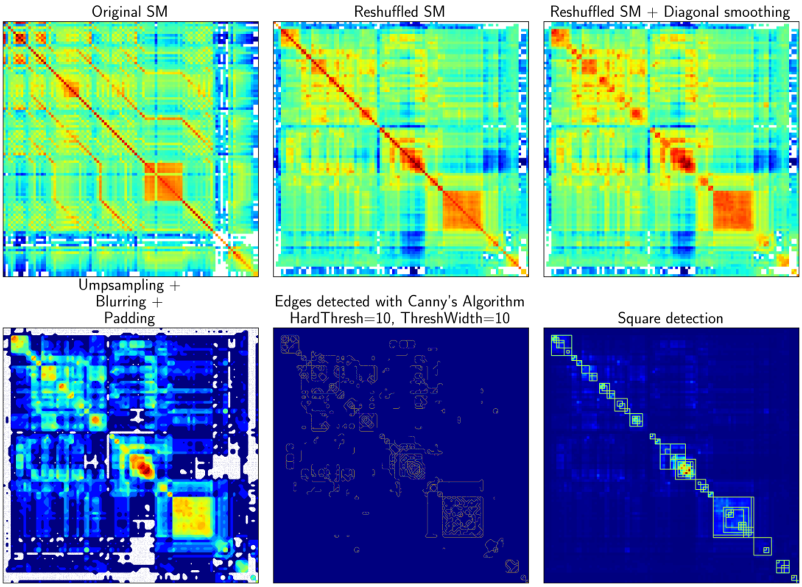

in reductions of usually > 50% in the number of families, indicating that in fact a large amount of raw such classes detected with computer vision vary significantly but, more importantly, that the noise reduction algorithm is able to detect and correct such results. This is even more pronounced during the second stage (step d in SI Fig. 5), where the total number of families is seen to reduce over typically 90%, resulting in 20 to 40 families per year.

Supplementary Note 6: Evolution of families of elements

Between 20 and 40 families were obtained for each year, some of which have non-empty intersections —in contrast to the standard periodic table, regarded as a system of partitions. Recurrent families include {Na, K, Rb, Cs}, {Ca, Sr, Ba}, and {F, Cl, Br, I}. To study their evolution, families are selected, that are consistently found over some period in history. Their development is tracked from 1800 to 2021 by comparing each selected family $\mathcal{F}$ against the families found for each year. For each family $\mathcal{F}$, and each year y, three cases

SI Fig. 7 Statistical noise reduction algorithm (SNR) for collections of sets. The goal of the algorithm is: given an input pool of collections of families of elements, produce a single collection of families, which results from considering the more statistically relevant features of the families in the input pool of collections. In a first step (***a***), for each set A_{ij} in collection C_i, a new collection $\{\sim A_{ij}\}$ is assembled, which contains the most similar sets found in every other input collection; similarity between sets is assessed through the Jaccard/Tanimoto similarity [10]. Such collection is then, in step ***b***, reduced to a single representative set, which contains every element that is contained in at least 50% of the sets in $\{\sim A_{ij}\}$. Such new representative set takes on the role of A_{ij}. For every newly created collection, all duplicate, empty or single-element families are removed (***c***). The result is a new pool of collection of families, which is then used as a new input a number M of iterations. In last step ***e***, all collections are merged into a single one, and duplicates, empty or single-element sets are removed again. The output is a single collection of sets, which captures the most representative features of the pool of collections given as input.

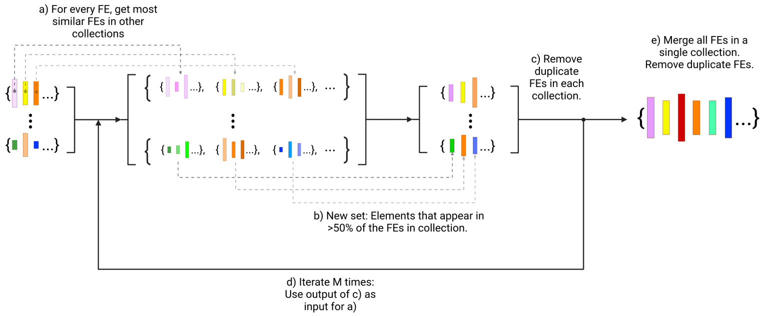

were considered: 1) $\mathcal{F}$ is found in y, 2) $\mathcal{F}$ is contained in another family found in y, and 3) None of these. Heat maps showing the results of such analysis are shown below, and a condense picture is given in Fig. 4 (Main text).

SI Fig. 8 Families of similar elements over time. Each family (index on the left) is searched in the collections of families of every year, and the colors indicate that the family: exists (black), is found as subset (green), or is not found (white) in the given year. Families are grouped together according to some key elements (shown on the right) they contain. Fig. 4 (Main text) shows a condensed version of this picture, where continuous bars represent the (sometimes only approximate) continuity of black blocks in this figure.

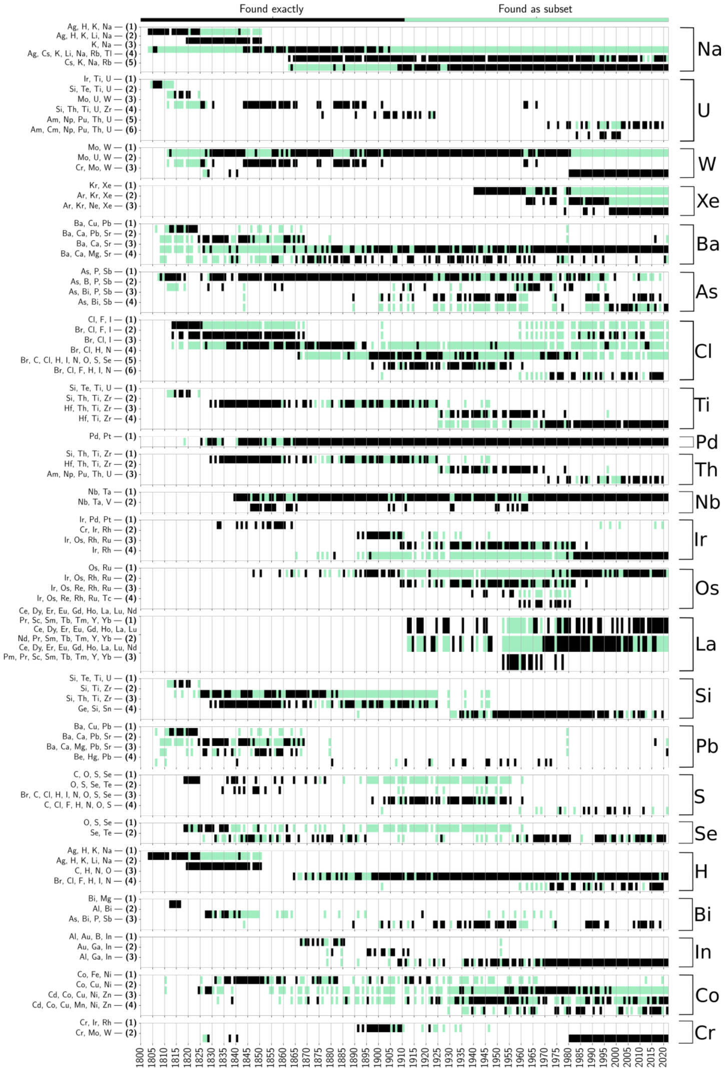

SI Fig. 9 Evolution in number of elements. Heatmap: Each cell (i, j) corresponds to $e_j - e_i$, where e_k indicates the number of elements in year k. Red values indicate low differences, while high values are indicated with blue colour. **Inset:** Number of elements each year.

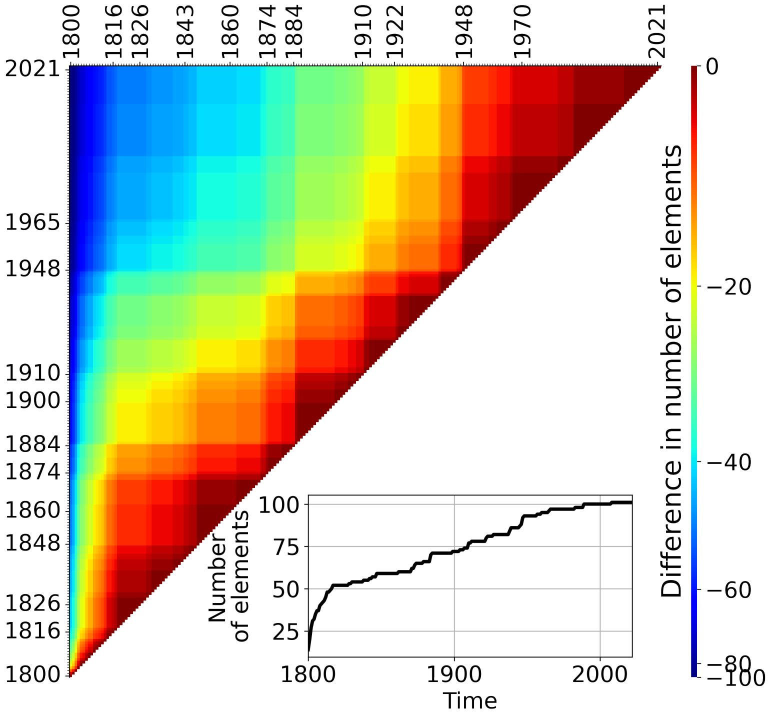

SI Fig. 10 The role of organometallic chemistry. For each transition metal, the templates found each year are filtered into: **Top.** Number of organometallic relations found for metals. Counted as the number of templates R—X_n in which X is metal (in row), and R contains C and some other organogenic element: H, N, O, F, Cl, Br, P, S. The values are normalised over the maximum of each row. **Bottom.** Number of non-organometallic relations found for metals. Counted as the number of templates R—X_n in which X is metal (in row), and R does not contain C. The values are normalised over the maximum of each row.

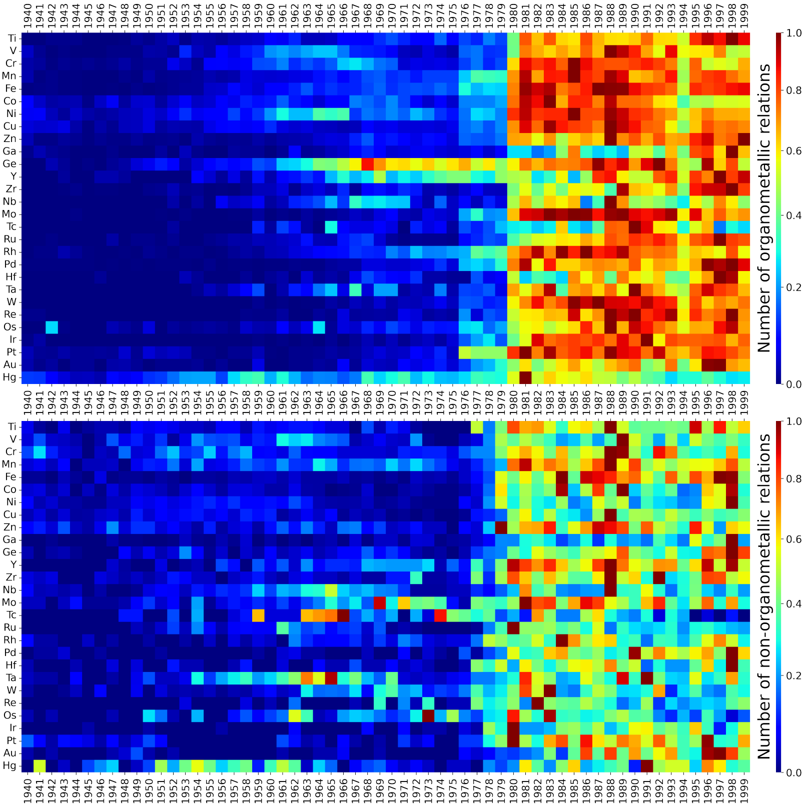

SI Fig. 11 Trends in composition of templates R—X$_n$, for X = actinoid. Relative amounts of templates that contain C or any other organogenic element in R (black), and those that do not (red).

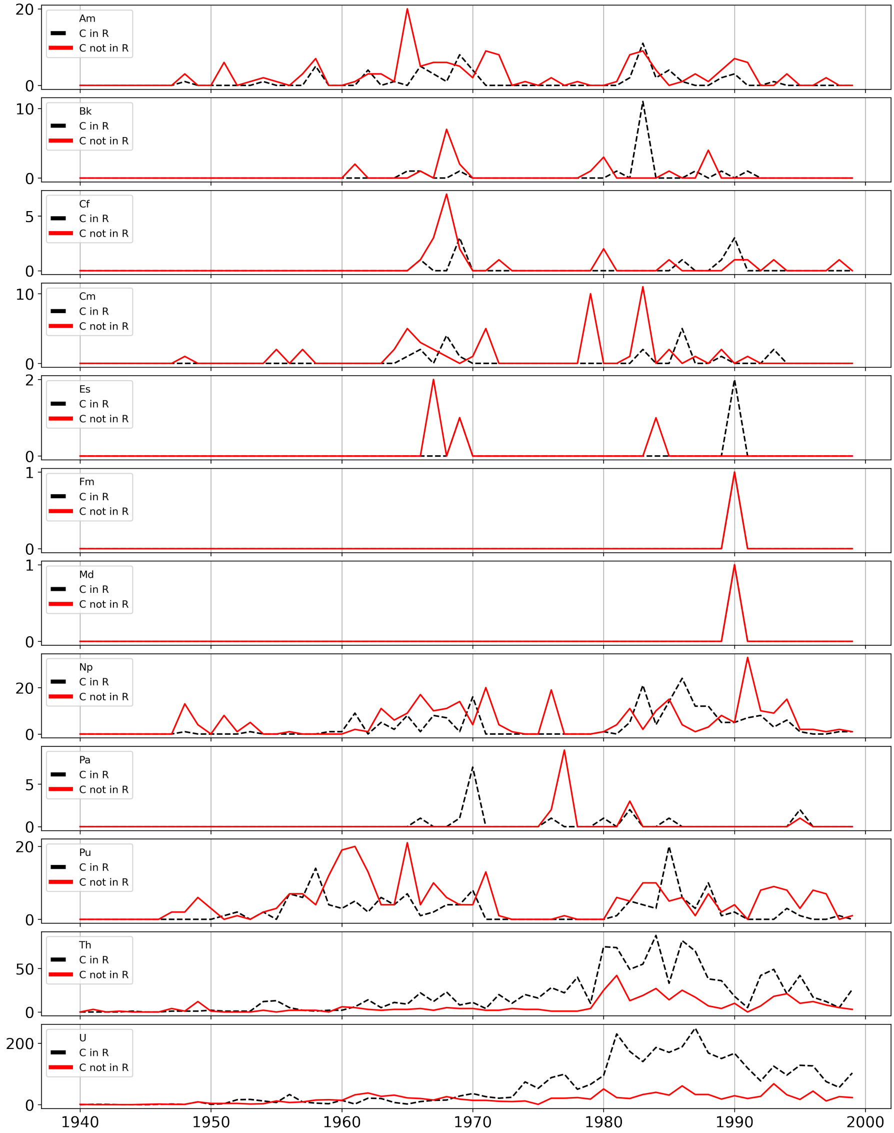

SI Fig. 12 Trends in composition of templates R—X$_n$, for X = lanthanoid. Relative amounts of templates that contain C or any other organogenic element in R (black), and those that do not (red).

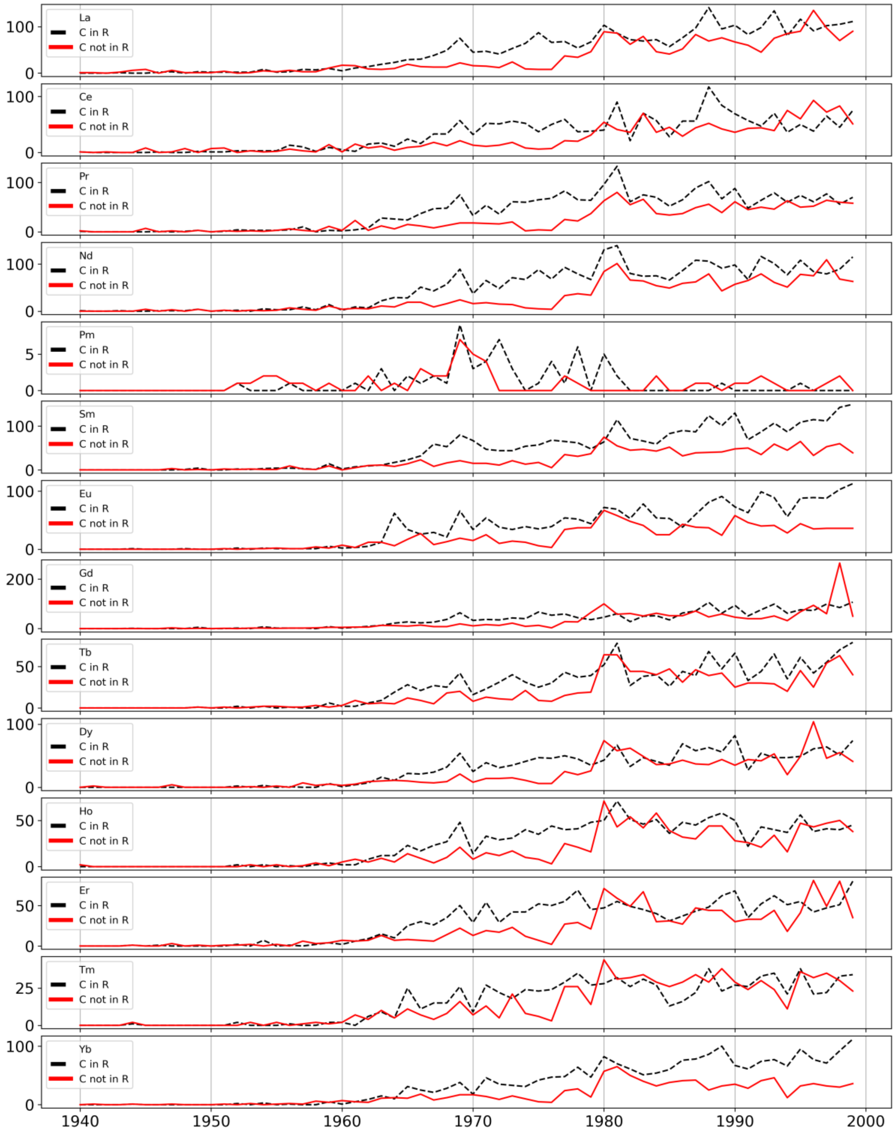

SI Fig. 13 Trends in composition of templates R—X$_n$, for X = {B, F, P, Si}. Relative amounts of templates that contain C or any other organogenic element in R (black), and those that do not (red).

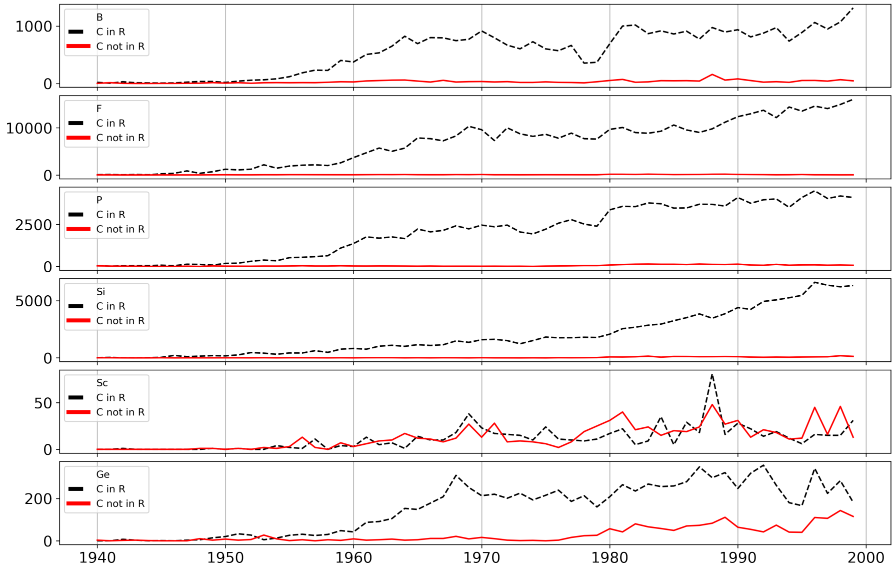

Supplementary References

[1] Elsevier. Reaxys database. URL https://www.elsevier.com/solutions/reaxys.

[2] Restrepo, G. Compounds bring back chemistry to the system of chemical elements. *Substantia* **3** (2), 115 – 124 (2019). URL https://riviste.fupress.net/index.php/subs/article/view/739. https://doi.org/10.13128/Substantia-739 .

[3] Llanos, E. J. *et al.* Exploration of the chemical space and its three historical regimes. *Proceedings of the National Academy of Sciences* **116** (26), 12660–12665 (2019). URL https://www.pnas.org/doi/abs/10.1073/pnas.1816039116. https://doi.org/10.1073/pnas.1816039116, https://arxiv.org/abs/https://www.pnas.org/doi/pdf/10.1073/pnas.1816039116 .

[4] Restrepo, G. Challenges for the periodic systems of elements: Chemical, historical and mathematical perspectives. *Chemistry – A European Journal* **25** (68), 15430–15440 (2019). URL https://chemistry-europe.onlinelibrary.wiley.com/doi/abs/10.1002/chem.201902802. https://doi.org/https://doi.org/10.1002/chem.201902802, https://arxiv.org/abs/https://chemistry-europe.onlinelibrary.wiley.com/doi/pdf/10.1002/chem.201902802 .

[5] Rayner-Canham, G. *The Periodic Table* (World Scientific, 2020). URL https://worldscientific.com/doi/abs/10.1142/11775. https://worldscientific.com/doi/pdf/10.1142/11775.

[6] Biron, E. V. The phenomenon of secondary periodicity (in Russian). *Zh. Russ. Fiz. Khim. Obshch* **47**, 964–988 (1915) .

[7] Pettifor, D. A chemical scale for crystal-structure maps. *Solid State Communications* **51** (1), 31–34 (1984). URL https://www.sciencedirect.com/science/article/pii/0038109884907658. https://doi.org/https://doi.org/10.1016/0038-1098(84)90765-8 .

[8] Glawe, H., Sanna, A., Gross, E. K. U. & Marques, M. A. L. The optimal one dimensional periodic table: a modified pettifor chemical scale from data mining. *New Journal of Physics* **18** (9), 093011 (2016). URL https://doi.org/10.1088/1367-2630/18/9/093011. https://doi.org/10.1088/1367-2630/18/9/093011 .

[9] Allahyari, Z. & Oganov, A. R. Nonempirical definition of the Mendeleev numbers: organizing the chemical space. *The Journal of Physical Chemistry C* **124** (43), 23867–23878 (2020). URL https://doi.org/10.1021/acs.jpcc.0c07857. https://doi.org/10.1021/acs.jpcc.0c07857, https://arxiv.org/abs/https://doi.org/10.1021/acs.jpcc.0c07857 .

[10] John David, M. & Norah E., M. *Clustering in Bioinformatics and Drug Discovery* 1st edn (CRC Press, London, 2011).
